# Quantitative Study on Reinforcing Mechanism of Nanofiller Network in Silicone Elastomer Based on Fluorescence Labeling Technology

**DOI:** 10.3390/polym16192829

**Published:** 2024-10-07

**Authors:** Yuquan Li, Yuqi Xiong, Ming Kang, Fengmei Yu, Ai Lu

**Affiliations:** 1 Institute of Chemical Materials, China Academy of Engineering Physics, Mianyang 621900, China; liyuquan@caep.cn (Y.L.); azong2021@mail.ustc.edu.cn (Y.X.); yufengmei@caep.cn (F.Y.); 2 State Key Laboratory of Environment-Friendly Energy Materials, Southwest University of Science and Technology, Mianyang 621010, China; kangm9690@163.com

**Keywords:** silicone elastomer, silica, fluorescence labeling, reinforcement model

## Abstract

Although there have been many theoretical studies on the enhancement effect of nanofiller networks and their interaction with elastomer molecular chains on the mechanical properties of elastomers, its mechanism description is still not completely clear. One of the main obstacles is the lack of quantitative characterization techniques and corresponding theoretical models for the three-dimensional morphology of complex nanofiller networks. In this paper, the precipitated silica-filled silicone rubber was studied by fluorescence labeling combined with laser scanning confocal microscopy, and the real three-dimensional images of dispersion and aggregation structure of filled rubber systems were obtained. The microstructure evolution of nano-particle aggregates caused by the increase in the filler volume fraction was quantitatively described, and the reinforcement mechanism of elastomers with a distribution of aggregates and filler networks composed of nanoparticles was studied. Furthermore, a nano-composite reinforcement model based on volume fraction, particle shape, interaction, and filler dispersion has been proposed.

## 1. Introduction

Silicone rubber has excellent high and low temperature resistance and aging resistance due to the organic-inorganic hybrid characteristics of its molecular chain, but its mechanical strength is poor without filler. The mechanical strength can be increased by tens of times after being filled with nanoparticles (NPs) [1,2,3,4,5]. However, the dispersion and packing behavior of the NPs are significantly affected by their own properties and processing methods, and it is necessary to regulate the contribution of filler–filler and filler–polymer interactions in the matrix to meet the requirements of different fields in practical applications. At present, numerous studies have demonstrated that the filler network enhances the performance of the material under large strain [6,7,8], but the improvement mechanical properties of the filled elastomer are still observed under the low filler load [9,10,11,12,13]. In addition, there is only a vague conclusion about the nonlinear mechanical mechanism of material with small amplitude strain under dynamic oscillation conditions. It is believed that the entanglement of molecular chains and the interaction between filler and polymer interface strengthen this behavior [14,15], but there is still a lack of intuitive and reliable quantitative proof. 

In the present theoretical models of composite materials, the dependence of performance enhancement on filler content has been widely expressed in mathematics. Smallwood proved that in rubber with dispersed rigid particles, both modulus and viscosity are dependent on concentration equivalently. Based on that, the Einstein–Smallwood equation (ESE) was established [16].

E* = E [1 + 2.5c](1)

where E* and E are the elastic modulus of filled rubber and pure rubber, respectively; c is the volume fraction of the filler. The research results show that the ESE equation has satisfactory prediction for the reinforcement of a rigid sphere dilution system. Rehner [17] applied the model to the calculation of certain elastic properties and highlighted the influence of the tensile strength of the stress concentration around the packing particles. Christensen [18] developed a geometric model of a macro-isotropic homogeneous matrix covering a single spherical particle to describe the reinforcement. Even in highly diluted systems, the interfacial interaction between rubber matrix and particles still exists. 

With the increase in the volume fraction of the filler, the spherical particles have contact with each other, and the filler aggregate develops, resulting in the filler network. Guth and Gold [19] developed the ESE equation by considering the interaction between particles in the packing concentration region (c = 0.1~0.3).

E* = E [1 + 2.5c + 14.1c^2^] (2)

They also took into account the influence of aggregation and believed that the spherical particles would converge to integrate the rod-like packing chain, so the parameters describing the shape were added, and the formula was further modified:

(3) $$E*=E\;(1+0.67\alpha \cdot c+1.62\alpha^{2}\cdot c^{2})$$

where α is the shape factor:

(4) α = length/width

Vikas Mitta [20] used a variety of traditional composite models to predict the tensile modulus of polyolefin nanocomposites. The results showed that traditional models could not describe this situation because the organic-inorganic interface of polyolefin nanocomposites did not have any positive interaction. Ouyang et al. [21] proposed a network junction model to study the energy dissipation process other than the slip of the packing surface and used this model to study the slip behavior between the packing surface and the rubber molecular chain in “closed rubber.” Aiming at the viscoelastic behavior of silicone rubber materials with large porosity under finite deformation, Zhanfang Liu et al. [22]. proposed a constitutive relationship suitable for the nonlinear viscoelastic mechanical properties of porous silicone rubber materials by establishing a relaxation function describing the viscoelastic characteristics of the materials, and the test results are in good agreement with the experimental data.

M.R.B. Mermet-Guyennet et al. [23] introduced the concept of “average particle size” to study the size dependence of enhanced properties of monodisperse polystyrene microsphere composites and supplemented and modified the ESE equation.

The above-mentioned models did consider the structure of the filler particles and their aggregates; however, the structure parameters are phenomenology and thus became fitting parameters. Due to the complex three-dimensional and multi-scale dispersion state of fillers in elastomer [24,25], how to truly characterize the dispersion state of fillers and coupling the microstructure and macroscopic properties of composites have been hot topics for researchers [26,27]. In recent years, the three-dimensional dispersion of carbon black in natural rubber under different strains was explored using synchrotron radiation, and the information about aggregate size and distance between aggregates of different components was obtained by rendering different components in composite materials [28,29]. Compared with conventional electron microscopy characterization methods, 3D X-ray imaging based on the synchrotron light source has the advantages of high spatiotemporal resolution; however, as a large scientific device, it is difficult to use it widely because of limited beam time accessed by a research group. In our previous study [30,31], we successfully used rare earth europium ions to label the surface of SiO_2_ and used laser scanning confocal microscopy (LSCM) to realize the visualization of fillers in silicone rubber, so as to master the evolution law of filler aggregates in the matrix under different filler contents. Without special processing of the sample, LSCM can directly obtain the image of the fluorescent phase (filler particles) and non-fluorescent phase (rubber matrix) in the filled rubber sample, intuitively and truly characterizing the dispersion state of the filler particles.

In this study, we investigate the origin of these performance enhancements by studying the mechanical behavior of silicone elastomer. Using the fluorescently labeled silica and LSCM, the real three-dimensional images of the dispersion and aggregation structure of filler particles (Ps) in silicone rubber were obtained, and the microstructure evolution of nano-particle aggregates caused by the increase in the filler volume fraction was quantitatively described. We extracted structure parameters from the in situ image results and improved the reinforcement model by considering the distribution of the filler particles (Ps). The fitting results support that the improved model describes the reinforcement of the elastomer better than the original one, which uses only particle average radius.

## 2. Experimental Section

### 2.1. Sample Preparation

Commercially available precipitated silica T36-5 (Shuanglong Chemical Co., Ltd., Tonghua, China) with a primary particle size of 20 nm were used for experimental investigations. Two kinds of methyl vinyl phenyl silicone gum (a kind of random linear co-polymer consisting of dimethylsiloxanes, diphenylsiloxanes, and mehtylvinyl siloxanes, abbreviated as PVMQ hereafter) with different phenyl contents (high-phenyl PVMQ, [2ph] = 25 wt % and low-phenyl PVMQ, [2ph] = 9.51 wt %, both obtained from Shandong University, Jinan, China) were selected as the rubber matrix. The surface fluorescence labeling of silica was carried out before mixing to meet the premise of LSCM. The surface treatment method and the preparation of filled rubber are shown in Supporting Information I.

The prepared sample name and volume fraction *φ_f_* are shown in Table 1.

Samples labeled as T-h were T36-5 silica-filled high-phenyl content PVMQ gum, and the number attached behind the “T-h” label represents the volume fraction of the T36-5 silica. For instance, T-h-18.52 represents a sample of high-phenyl content silicone elastomer reinforced with an 18.52% volume fraction of T36-5 silica. Similarly, samples labeled as T-l were T36-5 silica-filled low-phenyl content PVMQ gum, and the number attached behind the “T-l” label represent the volume fraction of T36-5 silica. For instance, T-l-4.35 represents a sample of low-phenyl content silicone elastomer reinforced with a 4.35% volume fraction of T36-5 silica.

### 2.2. Field Emission Scanning Electron Microscopy

The SEM images were obtained on the FESEM (Ultra55, Carl zeissNTS GmbH, Oberkochen, Germany) at an accelerating voltage of 10 kV after the sputter coating of gold on the specimen surface.

### 2.3. Laser Particle Analysis

A laser particle size analyzer (Brookhaven, New York City, NY, USA) was used to characterize the change of the silica particle size before and after the fluorescent labeling. A small amount of SiO_2_ powder was added into an appropriate amount of absolute ethanol, and it was poured into a cuvette for testing after ultrasonic dispersion.

### 2.4. Laser Scanning Confocal Microscope

The dispersion of fluorescently labeled silica in composites was observed by LSCM (Leica TCS Sp8, Leica, Wetzlar, Germany). The resolution of the confocal two-dimensional image is 1024 × 1024 pixels (voxel size: x = 0.568 μm, y = 0.568 μm, z = 0.148 μm). All images are in Tiff format, and the test time of 500 confocal two-dimensional images is about 25 min.

### 2.5. Measurement of Dynamic Mechanical Properties

The dynamic storage modulus G’ was measured with an RSA GII instrument using a control strain of 0.1%. This strain is in the linear response range confirmed by strain sweep.

### 2.6. 3D Visualization and Quantitative Analysis

**Figure 1:**
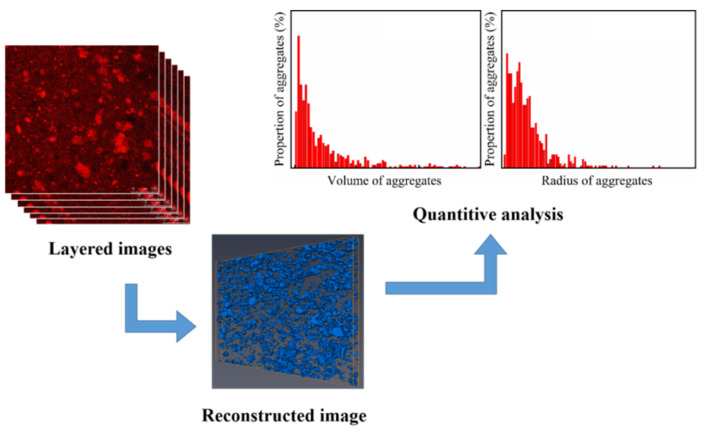
Three-dimensional visualization and quantitative characterization of fillers in silicone rubber. Red zone in the layered images represent the fluorescence labelled silica, and the blue zone in the reconstructed image represent the silica network formed by the silia aggregates.

To obtain the three-dimensional reconstruction picture, the noise of the 2D pictures was first eliminated. Since there are only two phases (fluorescently labeled silica phase and unlabeled rubber matrix phase) in the two-dimensional fluorescence image, it is easy to distinguish them. This operation is mainly used to adjust the threshold range of the image. The volume fraction *φ_f_* of fluorescently labeled silica in rubber under different threshold conditions was calculated by software, then the final threshold of fluorescently labeled silica was determined by matching the *φ_f_* with *φ_th_* (the volume fraction of fluorescently labeled silica in rubber calculated by formula). Through the operation of the volume-rendering module, the three-dimensional visualization image of the sample and the geometric parameters of the particles can be obtained at the same time (cf. Figure 1).

## 3. Results

### 3.1. Visualization Study on the Three-Dimensional Dispersion Structure of Silica Network

Given the utilization of two-dimensional micropictures to depict the dispersion size of the filler within the matrix results in distortion, it is statistically discernible that the result is notably smaller in comparison to the actual filler size; therefore, we choose LSCM and three-dimensional reconstruction technology to realize the visualization and quantitative characterization of filler dispersion. It has been proven in previous studies that the technology had little effect on the surface properties of silica, and the dispersion state obtained by LSCM will not be affected by fluorescence labeling [32,33]. 

**Figure 2:**
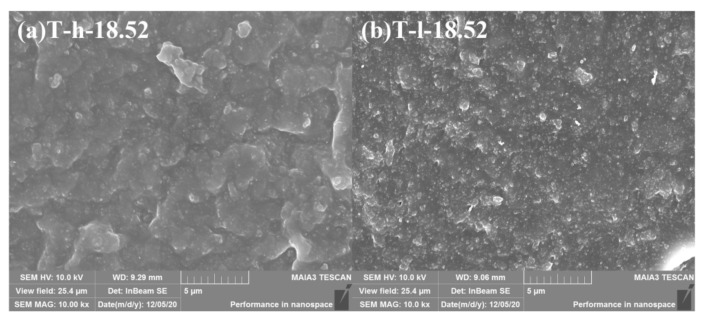
Dispersion characteristics of fluorescently labeled silica T36-5 filled-in silicone rubber (*φ_f_* = 18.52%) by FESEM.

**Figure 3:**
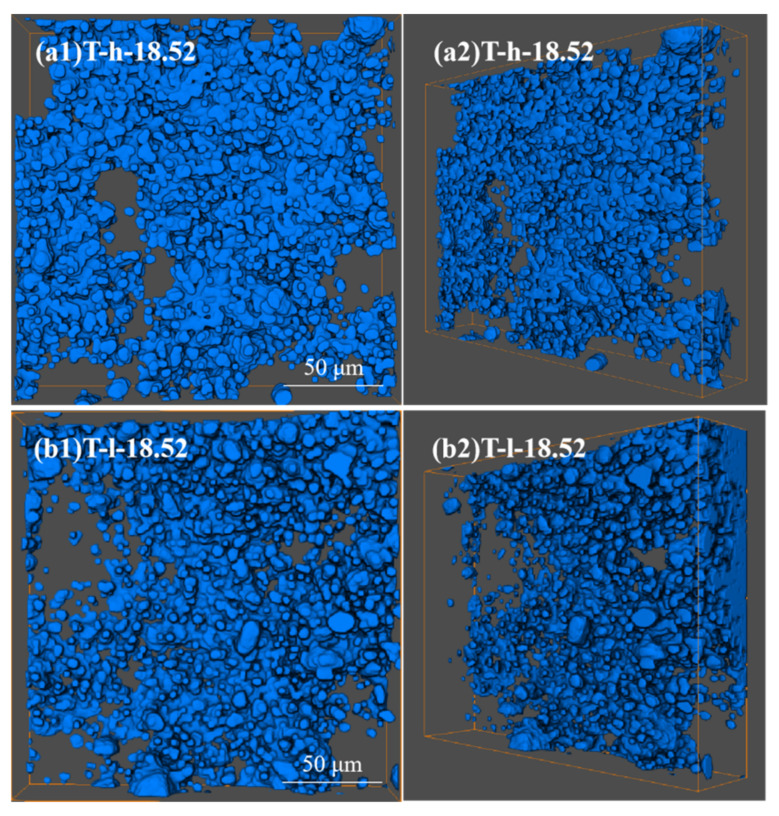
Dispersion characteristics of fluorescently labeled silica T36-5 filled-in silicone rubber (*φ_f_* = 18.52%) by LSCM.

**Figure 4:**
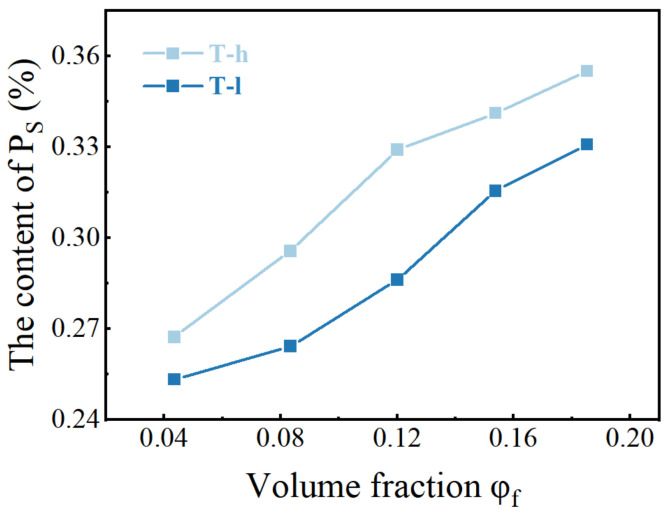
The content of P_S_ counted under the same reconstruction volume.

Different from Figure 2, the “cutting and decomposition” of rubber and particle groups can be realized by LSCM scanning and three-dimensional reconstruction technology, which shows the real spatial arrangement of particles (Figure 3). From the image, it can be seen that both PVMQ systems have high concentrations of particle agglomeration, but there are still small particles in the gaps, indicating that the fillers are not completely uniformly distributed in the matrix. In addition, the squint diagram shows many large aggregates with uneven distribution in the rubber matrix overlap. On one hand, the appropriate overlap promotes the formation of the network. On the other hand, excessive agglomeration size can lead to stress concentration and deteriorate the mechanical properties of the rubber matrix, which may lead to the difference in the performance of T-h and T-l systems. In order to quantitatively describe the enhancement mechanism of micro-dispersion on rubber properties, 4.35–18.52% volume fraction silica content rubber samples were reconstructed (Supporting Information II and III). The 3D images show that SiO_2_ presents a dense accumulation structure in silicone rubber with the increase in the filler loading, which is an intuitive manifestation of the small particles connecting into a continuous network. The radius of a primary silica particle is usually several nanometers, which cannot be distinguished by an optical microscope. Apparently, nano-scale primary silica particles aggregate into a larger structure. To discuss the structure of silica aggregates in the rubber, particles (Ps) were used, referring to the silica aggregates, while nano-particles (NPs) refers to the primary silica particles. Using the three-dimensional reconstruction software, the content of particles (P_S_) with the equivalent particle diameter less than 1 μm in different elastomers was counted, and the statistical volume range of each elastomer was shown in Figure 4. The results show that the increase in filler loading promotes the aggregation between NPs yet also increases the number of small particles. This is because the strong shear effect during mixing not only realizes the uniform dispersion of fillers in the matrix, it also improves the probability of internal friction between rigid particles. More importantly, this phenomenon is more significant under high filling. In this process, the continuously cut small particles are wrapped in the gap between the large particles and bridged by the soft matrix, which can effectively improve the spatial utilization of the dispersed medium. For all the filler contents, the T-h system always had higher contents of small particles. This is consistent with the SEM image results.

**Figure 5:**
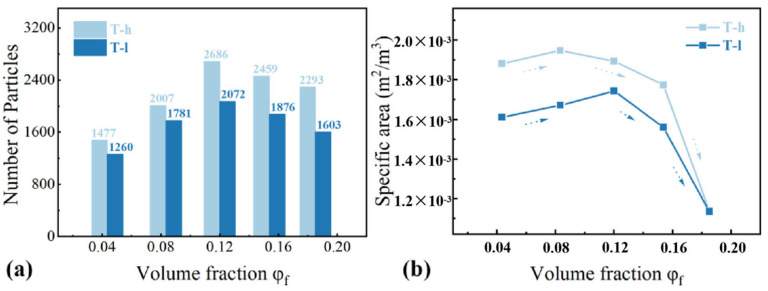
Characterization of spatial dispersion state of filler particles: (**a**) The number of distributed particles; (**b**) The specific surface area of particles in filled rubber with different volume fraction *φ_f_*: *S_NP_* = Σ*Ai*/Σ*Vi*, Σ*Ai* is the total surface area of aggregated silica particles, and Σ*Vi* is the total volume of aggregated silica particles. All statistics are conducted under the same reconstruction volume.

**Figure 6:**
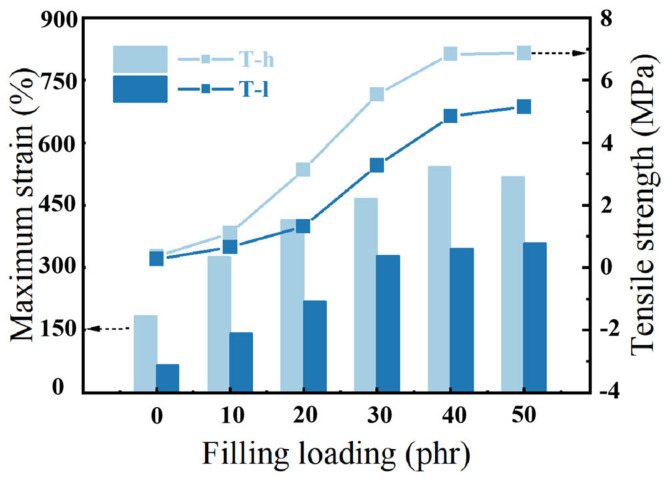
Engineering tensile strength and maximum strain curves at 0.83 mm/s of the PNCs.

The increase in the number and size of NPs will lead to the change of the three-dimensional network under sufficient filler concentrations. In order to explore the evolution of the filler dispersion state, the particle number and specific surface area of fillers under the same reconstruction volume were counted. The parameter *S_NP_* is defined as the actual specific surface area (surface area per unit volume). Mathematically, *S_NP_* is the ratio of total particle surface area (m^2^) and its total volume (m^3^), as expressed in formula *S_NP_* = Σ*Ai*/Σ*Vi*, in which Σ*Ai* is the total surface area of aggregated silica particles, and Σ*Vi* is the total volume of aggregated silica particles (cf. Figure 5). Three stages of agglomeration evolution are proposed to describe the development of SiO_2_ dispersion space structure with the increase in *φ_f_*: (i) In the first stage, the specific surface area and particle number of the filler increase gradually, and the filler is isolated in the matrix. (ii) As the filler content continues to increase (below the threshold), the filler particles gradually gather to form large-size aggregates. After the specific surface area and the number of particles gradually reached the maximum, the distance between aggregates decreased further. (iii) In the third stage, the connection between filler aggregates emerged, and gradually a network was formed, resulting in a decrease in particle number and specific surface area. The change in the filler aggregates structure with the increase in silica volume fraction has also be confirmed in other works [15]. Finally, the filler loading reaches the threshold when the filler network is established. Compared with elastomers without fillers, highly filled polymer composites better adapt to large strain without fracture and exhibit much better mechanical strength (Figure 6). The formation of the filler network and its contribution to the mechanical properties has been also confirmed by other types of fillers [34]. Through the above reasoning, it is speculated that the filler network is formed when the silica volume fraction is about 15.38~18.25%. The significant difference caused by phenyl content was observed again. The phenyl group has a larger volume and greater interaction between polysiloxane molecules, thus resulting in higher viscosity of the silicone gum. This leads to a stronger shear force when adding silica into the gum by banburying and better dispersion of the silica.

**Figure 7:**
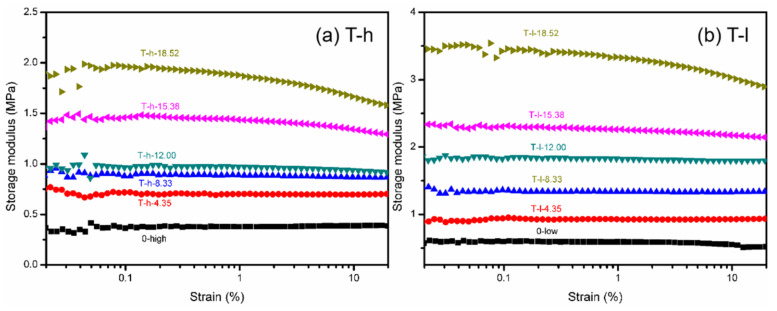
Storage modulus-strain curves at 20 °C, 1Hz of (**a**) T-h system and (**b**) T-l system.

The change in the storage modulus of PVMQ samples under large strain amplitude (cf. Figure 7) is a quantitative characterization of the “filler network” formed in the process of rigidly filled nanoparticles as solid infiltration in composites. With the increase in strain amplitude, the filler network breaks, and the modulus decreases. Therefore, both PVMQ systems show the existence of the filler network when the silica volume fraction is above 12.00%, reflecting the enhancement of elastomer (*φ_f_* = 4.35~8.33%) first attributed to the outstanding contribution of the small local filler network and filler–polymer interaction. The strain scanning results show that the percolation value of this SiO_2_ is about *φ_f_* = 12.00%, and the mechanical reinforcement of the composite is consistent with the three-dimensional dispersion evolution of the filler.

### 3.2. Discussion of Reinforcement Models

In order to further illustrate the effect of filler content on rubber properties, reinforcement models are discussed and compared. The normalized equation describing it is defined as follows:

(5) $$R=\frac{G\left(\varphi_{f}\right)}{G\left(\varphi_{f}=0\right)}-1$$

**Figure 8:**
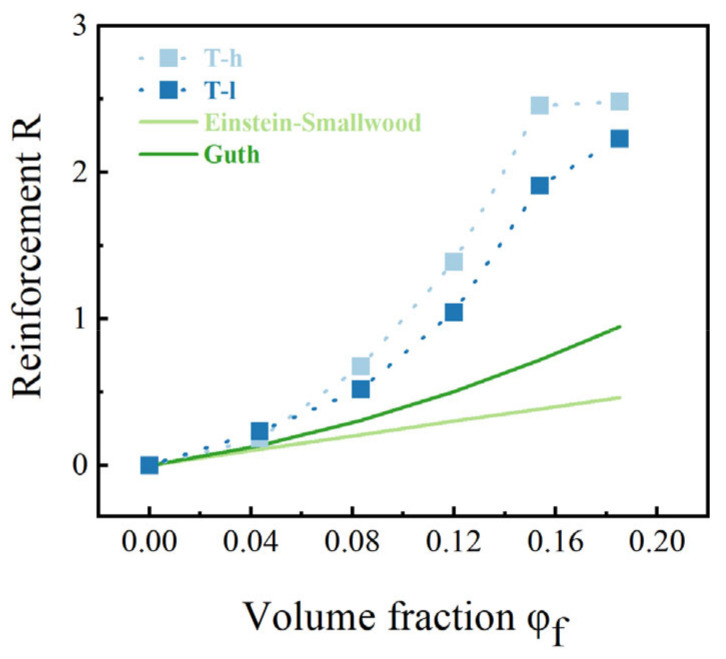
Reinforcement versus volume fraction *φ_f_* of the composites systems, obtained from static tensile experiments. The light and dark green solid lines correspond to the predictions of the Einstein−Smallwood and Guth models, respectively.

Young’s modulus at 1% strain was selected to describe the reinforcement, which is obtained by the slope of the stress-strain curve (cf. Figure S6). The enhancement factor R reflects a strong volumetric dependence of the filler (cf. Figure 8). By comparing the serious deviation of this research system from the ESE equation and the Guth equation under high filling, it is shown that under sufficient filling concentration, the influence of complex interaction in the composite elastomer is much higher than that of the strengthening of the hydrodynamic effect, and the model with *φ_f_* as the sole variable cannot define the reinforcement. Therefore, we need a mathematical model that can simultaneously consider multiple factors to describe the multi-scale reinforcement mechanism.

Mermet-Guyennet et al. [35] proposed a model based on a series of Gaussian transformations on the ESE equation and proven by experiments, which considered the reinforcement results of composites predicted by volume fraction *φ_f_* and average particle size *r_ave_*:

(6) $$R=2.5\varphi_{f}+C\varphi_{f}^{3}/r_{ave}$$

In the above equation, the linear term reflects the reinforcement of the filler volume effect, and the nonlinear term represents the interaction between the matrix and filler particles. This interaction comes from a chain that must form a continuous path between the boundaries of the filler particles, which encapsulates the particles as a whole to form a phase interface or provides a bridge connection between NPs. In this interaction model, the main factors involved are the force strength and the content of the interface. Since C in this model reflects only the physical parameters related to the properties of filler and matrix, it can be used as a quantitative characterization of the interfacial interaction strength.

*r_ave_* is the average particle size of dispersed nanoparticles in the system for a certain volume fraction. In this study, the “dilution method” was used to prepare low-fraction PNCs by diffusing the well-distributed filled rubber into the unfilled raw gum. In this process, the fillers were not directly dispersed into the rubber but as aggregates wrapped by the polymer chain, and the agglomeration of the fillers was thus weakened. It can be seen that in the mixing process, the dispersion state of fillers will be changed by the differences in filler content, mixing method, and rubber matrix. In these situations, the distribution state of the nanoparticles is different, which is difficult to summarize with the average particle size.

Therefore, we consider calibrating the average particle size with the particle dispersion, so that the model can more truly describe the effect of filler dispersion on the reinforcement of the elastomer. Macroscopically, the dispersion of fillers is the addition of each particle dispersion:

(7) $$R=2.5\varphi_{f}+C\varphi_{f}^{2}\times \sum_{i=1}^{n}\frac{\varphi_{i}}{r_{i}}$$

(8) $$\sum_{i=1}^{n}\varphi_{i}=\varphi_{f}$$

In the upper equations, the average radius of filler particles was replaced by a normalized radius considering the dispersion of particle size. In comparison with Equation (6), the linear term remains the same, which reflects the volume effect of filler particles. The non-linear term still reflects the interaction between filler particles and matrix, but the dispersion of particle size was considered. Filler particles, regardless of size, interact with the matrix via the interface layer. *Cφ_f_*^2^ is an interaction term related to the choice of the system, and $\frac{\varphi_{i}\;}{r_{i}}$ refers to the contribution of aggregates with volume fraction *φ_f_* and radius *r_i_* to rubber reinforcement. *D_i_* is defined as the ratio of the volume of particle *i* to the total volume of the particle, namely,

(9) $$D_{i}=\frac{\varphi_{i}}{\varphi_{f}}$$

(10) $$\sum_{i=1}^{n}D_{i}=1$$

Then, Equation (6) is equivalent to

(11) $$R=2.5\varphi_{f}+C\varphi_{f}^{3}\times \sum_{i=1}^{n}\frac{D_{i}}{r_{i}}$$

Equation (11) is a mathematical expression that describes the particle distribution effect and reinforcement correlation based on the average particle size theory and can also be regarded as the cubic term *φ_f_*^3^ correction of the ESE relationship multiplied by the specific surface area *S_NP_* of the filler (surface area per unit volume) *S_NP_* = 3Σ*D_i_/r_i_*, i.e., *R*~*φ_f_*^3^*S_NP_*. The shape factor (length/width) was simply fitted. Specifically, in the regime of matrix entanglement, the storage modulus at ω = 100 rad/s (cf. Figure S7) follows the Guth equation [18]:

(12) $$G^{’}=G_{\mathrm{matrix}}^{’}[1+0.67\alpha \varphi_{f}+1.62(\alpha \varphi_{f}{)}^{2}]$$

where *α* is the shape factor (length/width), and the fitting results show that the α of T36-5 is about 6~7. Since the shape factor of an ideal sphere is 1, the obtained *α* value of T36-5 indicated the deviation of the assumption that the aggregated silica particles are a sphere from the real situation. According to Sanat K. Kumar et al. [34], the reason why the shape factor deviates from the ideal value is that the NPs of primary silica particles form the aggregates by loosely packing with each other, instead of forming a solid sphere. In fact, the simplification that filler aggregation particles form a sphere makes the volume and centroid of each equivalent sphere equal to the corresponding SiO_2_ solid spherical particles, and the shape factor will then be greatly different from the actual situation. Thus, the actual specific surface area, defined as *S_NP_* in Section 3.1, is used to replace the Σ*D_i_/r_i_* to describe the contribution of the interaction between matrix and filler particles to reinforcement. Then, Equation (11) is equivalent to

(13) $$R=2.5\varphi_{f}+\frac{1}{3}C\varphi_{f}^{3}S_{NP}$$

**Figure 9:**
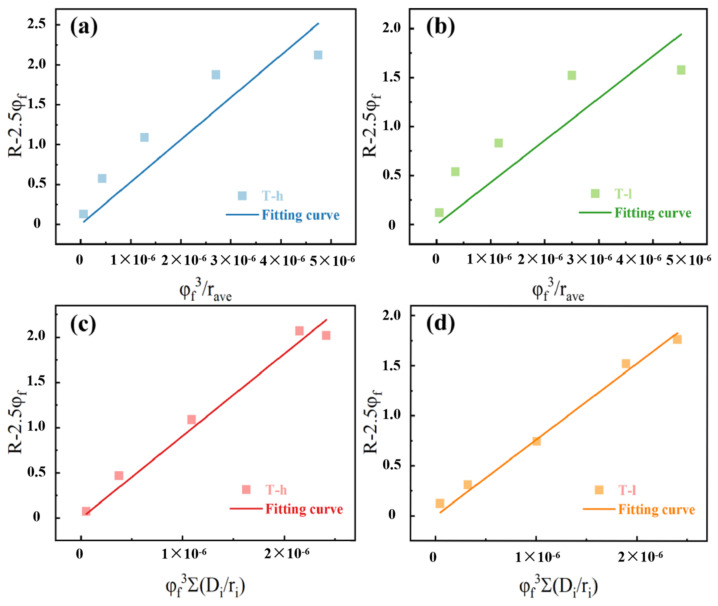
The filler distribution dependence of the reinforcement R of the composites systems: dependent of *R*-2.5*φ_f_* on *φ_f_^3^/r_ave_*: (**a**) T-h system and (**b**)T-l system; dependent of *R*-2.5*φ_f_* on *φ_f_*^3^Σ (*D_i_/r_i_*): (**c**) T-h system and (**d**) T-l system.

**Figure 10:**
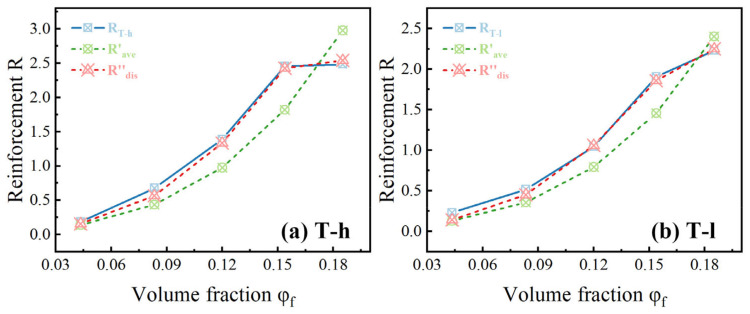
Reinforcement results redefined by the value of *C*: (**a**) T-h system and (**b**) T-l system; the black solid line is the true value of reinforcement *R*, the blue dotted line is the fitting result of average particle size *R*’*_ave_*, and the red dotted line is the fitting result of particle distribution *R*″*_dis_*.

In order to prove the advantage of particle distribution replacing average particle size in predicting rubber mechanical strengthening, the obtained data for *R*-2.5*φ_f_* were plotted as a function of *φ_f_*^3^/*r_ave_* and *φ_f_*^3^Σ(*D_i_/r_i_*), respectively. At least in the concentration range involved in this study, a straight line should be obtained. The fitting of particle distribution has a stronger linear correlation (cf. Figure 9a,b), while the fitting of average particle size shows an unreasonable arc because it ignores the influence of this factor on the interfacial adhesion (cf. Figure 9c,d). For this difference, it can be more intuitively shown in the comparison curve of the secondary simulation of *R* using the fitting result *C* (cf. Figure 10). *r_ave_* can only mechanically describe the exponential dependence of enhancement on filler concentration, while the abnormal enhancement caused by the mixing method is obviously consistent with the fitting curve of particle dispersion when *φ_f_* = 18.52%. Therefore, we conclude that the value obtained by linear fitting is *C* = 1 mm in the T-h system, and *C* is 0.7 mm in the T-l system.

To summarize, the simplification of the dispersion of particle distribution by using average radius [23] brought a deviation from the actual situation since the obtained shape factor is much larger than that of an ideal sphere. We improved the reinforcement model by considering the distribution of filler particle size, and this improvement brought better fitting results.

## 4. Conclusions

The influence of the dispersion distribution state of the filler in the matrix is certain, but the parameters describing the filler dispersion are lacking in the relevant enhancement model, which is studied in detail in this paper. Using the fluorescently labeled silica and LSCM technology, the 3D image data of silica dispersion in silicone rubber were obtained, the composite material enhancement model, including the filler volume fraction, the interaction between filler and matrix, and the filler dispersion parameters, was successfully constructed, and the validity of the model was verified by experimental data.

## Figures and Tables

**Table 1:** Constituents and volume fraction *φ_f_* of the samples.

	Label	Sulfur (phr)	Fluorescent Silica T36-5 (phr)	$\varphi_{f}$ (Vol %)
Unfilled high-phenyl PVMQ ([2ph] = 25%)	0-high	1.5	0	0
Unfilled low-phenyl PVMQ ([2ph] = 9.51%)	0-low	1.5	0	0
High-phenyl PVMQ filled with T36-5	T-h-4.35	1.5	10	4.35
	T-h-8.33	1.5	20	8.33
	T-h-12.00	1.5	30	12.00
	T-h-15.38	1.5	40	15.38
	T-h-18.52	1.5	50	18.52
Low-phenyl PVMQ filled with T36-5	T-l-4.35	1.5	10	4.35
	T-l-8.33	1.5	20	8.33
	T-l-12.00	1.5	30	12.00
	T-l-15.38	1.5	40	15.38
	T-l-18.52	1.5	50	18.52

## Data Availability

The original contributions presented in the study are included in the article/Supplementary Materials; further inquiries can be directed to the corresponding authors.

## Supplementary Materials

The following supporting information can be downloaded at: https://www.mdpi.com/article/10.3390/polym16192829/s1, Figure S1: Thermal gravimetric curve of silica T36-5 after and before fluorescent labeling; Figure S2: Particle size distribution curve of T36-5 after and before fluorescence labeling; Figure S3: Three-dimensional restructure of fluorescent-labeled silica in silicone Rubber for (a1)~(a5) and (b1)~(b5): T-h system; (c1)~(c5) and (d1)~(d5): T-l system; Figure S4: Aggregate volume distribution map of fluorescent-labeled T36-5 with φf = 4.35%~18.52% in silicone rubber. (a1)~(a5) T-h system; (b1)~(b5) T-l system; Figure S5: Aggregate size distribution map of fluorescent-labeled T36-5 with *φ_f_* =4.35%~18.52% in silicone rubber. (a1)~(a5) T-h system; (b1)~(b5) T-l system. The statistical particle size in the figure is the equivalent radius (*r_i_* = $\frac{1}{2}\sqrt[{3}]{\frac{6V}{\pi }}$); Figure S6: Stress-strain curves of (a) T-h system and (b) T-l system with *φ_f_ =* 4.35~18.52%. The tensile rate is 0.83mm/s, and the test is performed at 25 °C; Figure S7: Storage modulus recorded as a function of angular frequency (ω) at 25 °C for (a) T-h system and (b) T-l system. The storage modulus at ω = 100 rad/s can be used to simply quantify the shape factor of the filler particle; Figure S8: Loss modulus-strain curves at 20 °C, 1Hz of (a) T-h system and (b) T-l system.
