# Supplementary material for: Quantitative Study on Reinforcing Mechanism of Nanofiller Network in Silicone Elastomer Based on Fluorescence Labeling Technology
Source: Polymers (Basel). 2024 Oct 7;16(19):2829. doi: 10.3390/polym16192829 (PMC11478360; doi:10.3390/polym16192829)
Supplement: Supplementary file 1 [file polymers-16-02829-s001.zip › polymers-3174811-supplementary.pdf]

Supporting Information

I. Materials and Methods

Precipitated silica (T36-5, Tonghua Shuanglong Chemical Industry Co., Ltd.) with a primary particle size of 20 nm was used for experimental investigations. It was mixed into different rubber, i.e., methyl vinyl phenyl silicone rubber (25% biphenly content, 0.53% vinyl content) and methyl vinyl phenyl (9.51% biphenly content, 0.47% vinyl content), respectively. Benzoyl peroxide (BPO, AR) as a vulcanizing agent was purchased from Shanghai Merrill Chemical Technology Co., Ltd. Europium(III) nitrate hexahydrate ($Eu(NO_3)_3 \cdot 6H_2O$, 99.99 %), propyltriethoxysilane isocyanate (ICPTES, 95 %), and dibenzoylmethane (DBM, AR) used in the synthesis process were purchased in Shanghai Aladdin Biochemical Technology; the tetrahydrofuran (THF, AR) and sodium hydride (NaH, AR) were purchased from Shanghai McLin Biochemical Technology.

The T36-5 were fluorescently labeled according to our previous work[1] before padding into rubber. First, 2 mmol DBM were weighed as the reactant, and 2 mmol NaH were added as the catalyst, and a 40 ml THF solution was added as the solvent into the flask. The reaction temperature was controlled at 60 °C for 2 h in N_2 atmosphere. An amount of 2 ~ 8 mmol ICPTES was absorbed by a needle tube and then added to three flasks by drip through a constant pressure drop funnel. The mixture was stirred for another 4 h under N_2 protection. After the reaction was completed, the appropriate amount of 300 ~ 400 mesh silica gel powder was added to the crude product and mixed uniformly. The product was separated and purified by silica gel column chromatography. The red-brown oily liquid obtained after rotary evaporation is a bifunctional silane coupling agent. Next, the synthesized bifunctional silane coupling agent was used as a "bridge" to label the T36-5. An amount of 0.3 mmol DBM-Si was accurately weighed and placed in a 50 ml single flask A, and 10 ml anhydrous ethanol was added to dissolve it. An amount of 0.1 mmol $Eu(NO_3)^3 \cdot 6H_2O$ was placed in a 50 ml single-port flask B and dissolved in 3 ml anhydrous ethanol. The solution in the single flask B was dripped into the A flask, and the reaction temperature was 50 °C, PH = 7, with constant temperature and stirring for 30 min. The product was a light yellow clarification liquid, which was named as $Eu(DBM\text{-}Si)_3$. An amount of 4 g silica was placed in 80 ml ethanol and deionized water (ethanol : water = 1 : 1), with ultrasonic dispersion for 10 min. The $Eu(DBM\text{-}Si)_3$ was added into the silica dispersion solution by drop through a constant pressure drop funnel, and the reaction temperature was controlled at 50 °C for 2 h. After the reaction was completed, the obtained solution was centrifuged at a high speed for 5 min to retain the solid product and then washed with deionized water and anhydrous ethanol five times. The final white product was dried in an oven at 80 °C for 24 h to obtain fluorescently labeled SiO_2. The content of $Eu(DBM\text{-}Si)_3$ was 13 wt%, and the fluorescent-labeled silica was named $Eu(DBM\text{-}Si)_3@SiO_2$.

The silicone rubber filled with SiO_2 (the filler content was 60 phr, $\varphi_f = 21.43$ %) was prepared by torque rheometer (RC400P, HAAKE co., Ltd., Germany). Using the general mixing steps, the raw rubber was first added into the mixing chamber and stirred for 5 minutes at a rotating speed of 60 rpm and a temperature of 115 °C, so that it was fully preheated and filled with the chamber. Then, the 60 phr fluorescent-labeled silica was divided into three parts on average, and one part was added to the chamber every 5 minutes. After mixing for another 10 minutes, the filler was removed and remelted for 15 minutes, so that the filler was fully mixed in the rubber matrix. Silicone rubber filled with other content of silicon dioxide was prepared by adding 60 phr mixed rubber into the open homogenizer with the corresponding mass of silicone rubber diluted at 10 rpm. The prepared filled silicone rubber was placed at room temperature for 3 days and vulcanized at 115 °C for 30 min.

II. Effect of Fluorescent Labeling on White Carbon Black

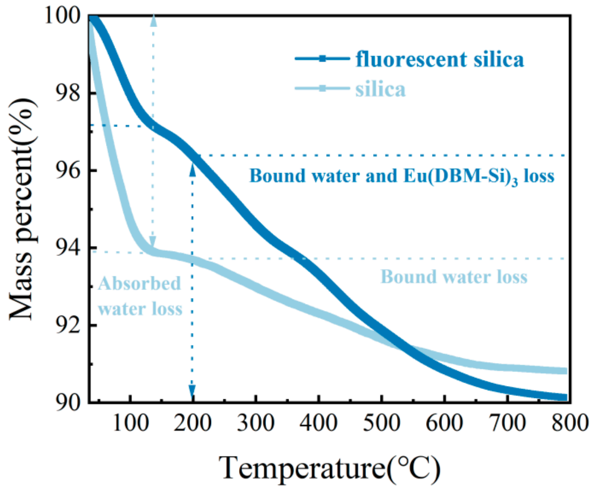

Figure S1 Thermal gravimetric curve of silica T36-5 after and before fluorescent labeling

Figure S1 shows that thermogravimetric curve of T36-5 before and after fluorescence labeling. The weight loss peak below 200 °C is attributed to the thermal decomposition of water adsorbed on the surface of SiO_2. Compared with the sharply reduced mass of pure SiO_2, the water decomposition content of fluorescent labeled SiO_2 is less, indicating that the synthesis process consumes part of the silicon hydroxyl on the surface of silica and improves the hydrophobicity of the filler. When the temperature is higher than 200 °C, the weight loss of fluorescent-labeled SiO_2 is higher. The weight loss of pure silica is mainly due to the removal of bound water on the filler surface, while the weight loss of fluorescent-labeled silica includes the thermal decomposition of bound water and organic matter. By comparing the residual mass at a high temperature, it can be concluded that the weight loss difference before and after fluorescent labeling is

very small (less than 1 %), that is, the fluorescent labeling method used in the experiment can achieve the effect of fluorescent labeling only by consuming very little silicon hydroxyl on the surface of SiO_2 and has little effect on the surface properties of the filler.

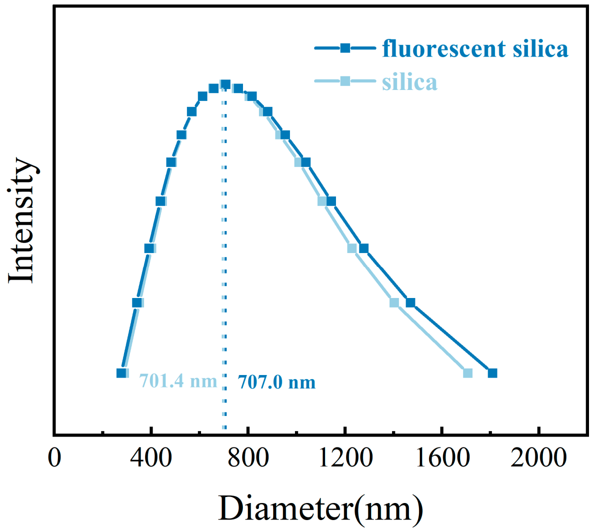

Figure S2 Particle size distribution curve of T36-5 after and before fluorescence labeling

Fig. S2 shows the dispersion state of pure T36-5 and fluorescent-labeled T36-5 in absolute ethanol. It can be seen that the synthesis process has little effect on the surface properties of fillers even if the surface hydroxyl groups are consumed. In addition, the aggregate size of T36-5 in ethanol is much larger than its primary particle size of 20 nm, indicating the polydispersity of the filler.

III. 3D Visualization and Quantitative Characterization of Dispersion

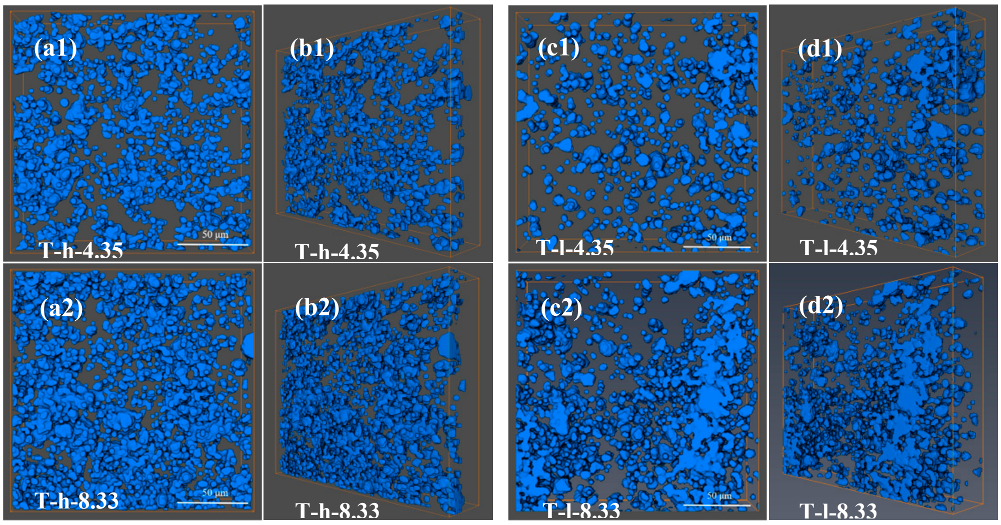

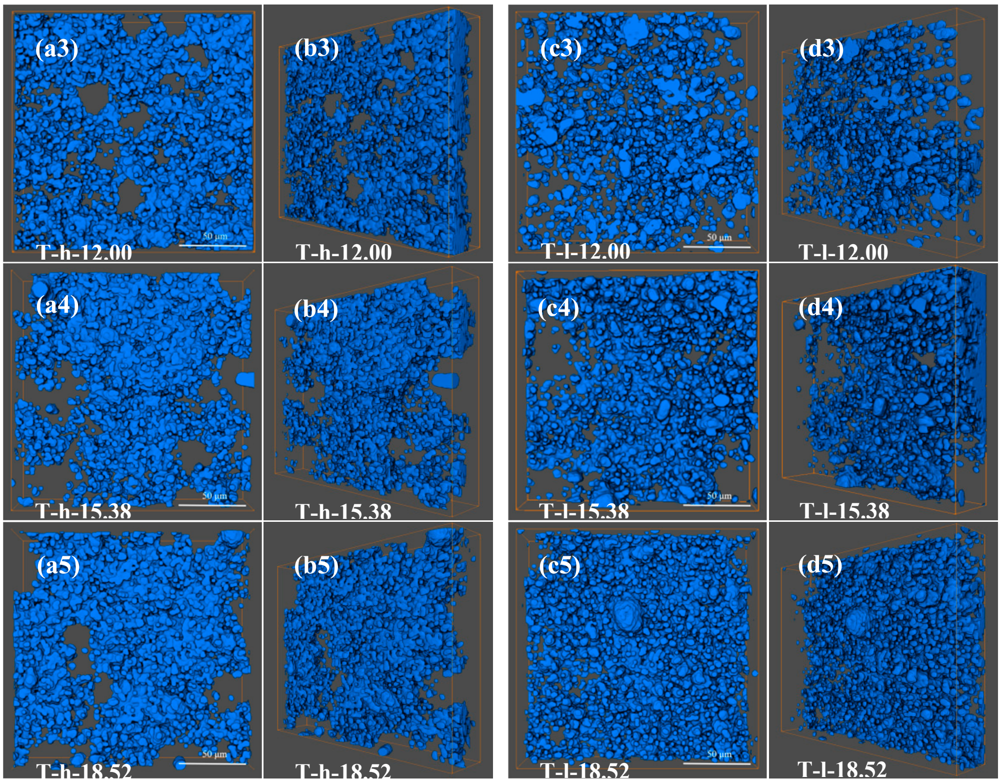

Figure S3 Three-dimensional restructure of fluorescent-labeled silica in silicone Rubber for (a1) ~ (a5) and (b1) ~ (b5): T-h system; (c1) ~ (c5) and (d1) ~ (d5): T-l system.

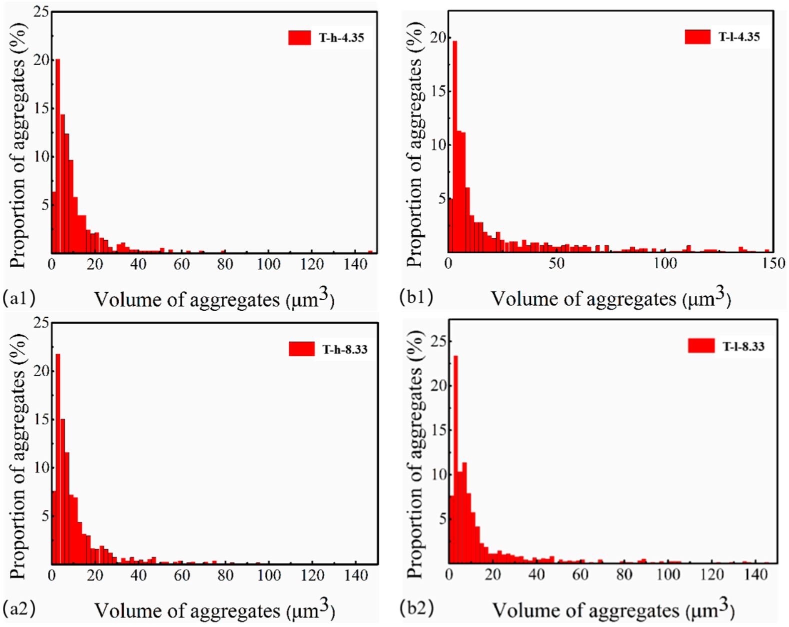

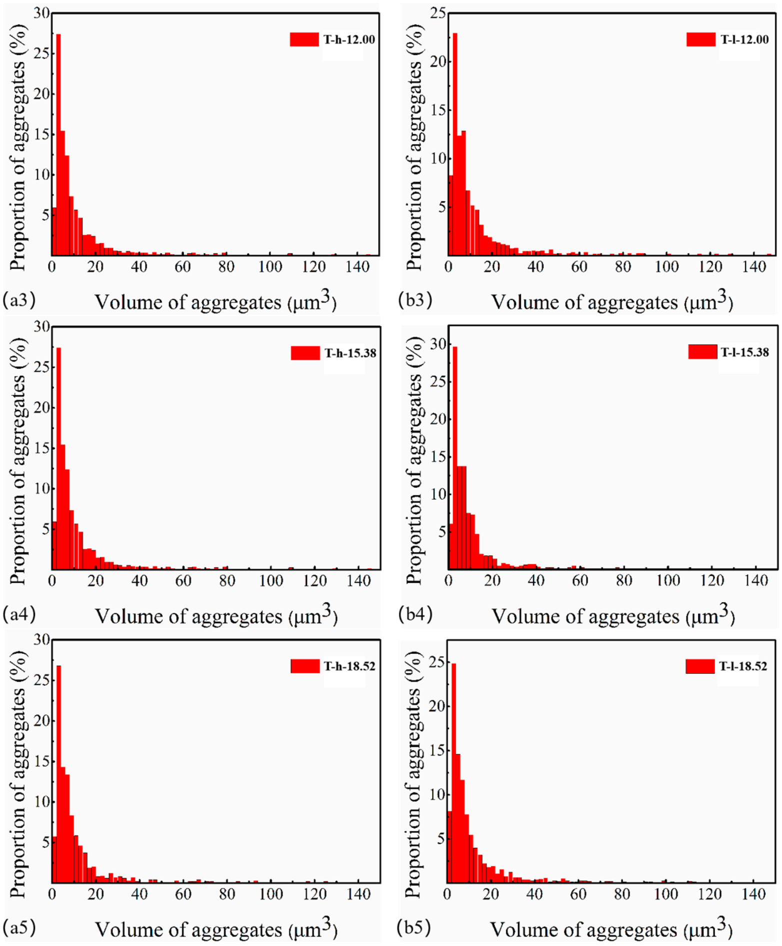

Figure S4 Aggregate volume distribution map of fluorescent-labeled T36-5 with φ_f = 4.35% ~ 18.52% in silicone rubber. (a1) ~ (a5) T-h system; (b1) ~ (b5) T-l system.

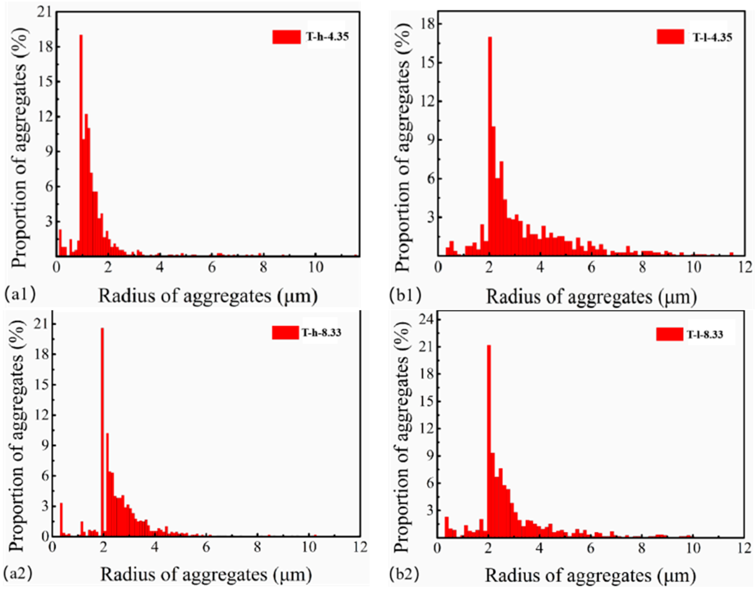

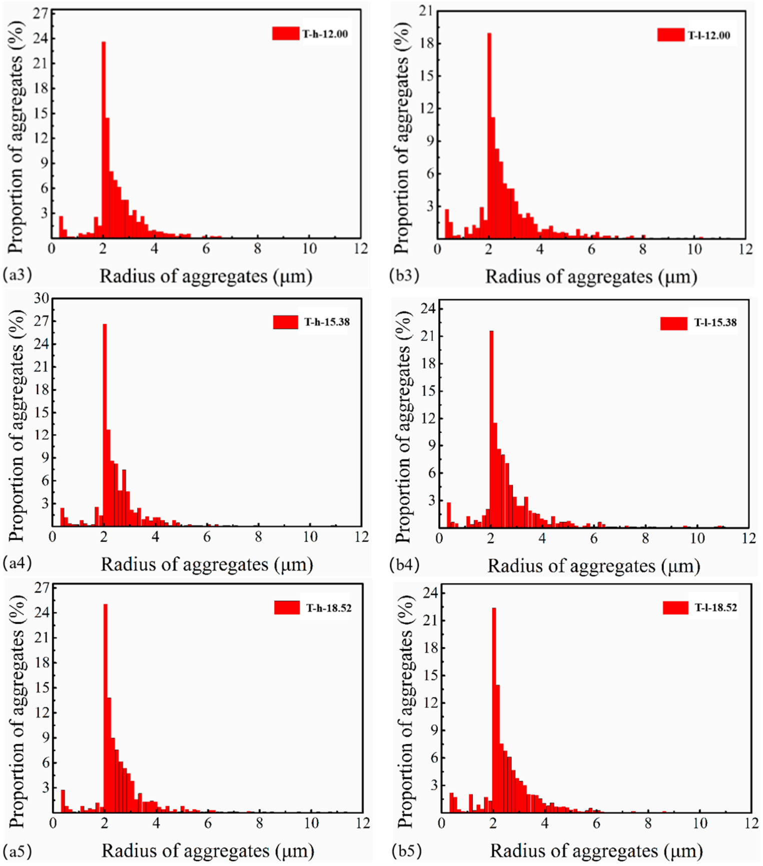

Figure S5 Aggregate size distribution map of fluorescent-labeled T36-5 with φ_f =4.35% ~ 18.52% in silicone rubber. (a1) ~ (a5) T-h system; (b1) ~ (b5) T-l system. The statistical particle size in the figure is the equivalent radius ($r_i = \frac{1}{2}\sqrt[3]{\frac{6V}{\pi}}$).

IV. Mechanical Properties Test of Composite Rubber

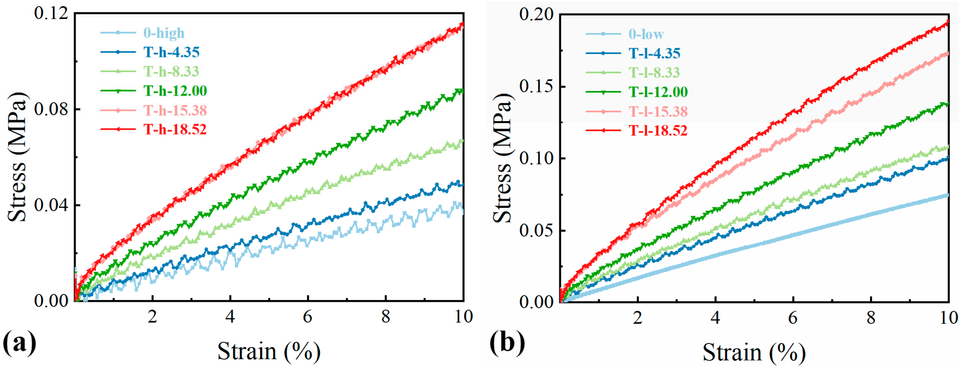

Figure S6 Stress-strain curves of (a) T-h system and (b) T-l system with φ_f = 4.35 ~ 18.52%. The tensile rate is 0.83mm/s, and the test is performed at 25°C.

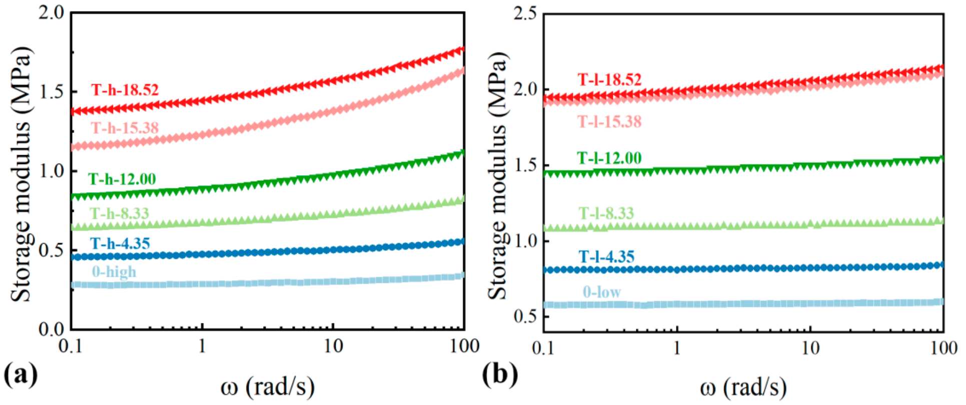

Figure S7 Storage modulus recorded as a function of angular frequency (ω) at 25 °C for (a) T-h system and (b) T-l system. The storage modulus at ω = 100 rad/s can be used to simply quantify the shape factor of the filler particle.

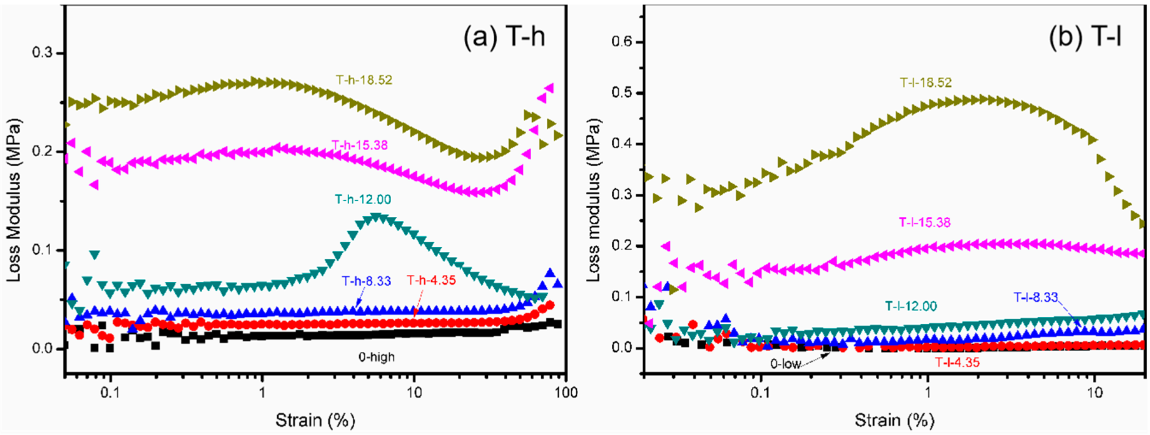

Figure S8 Loss modulus-strain curves at 20 °C, 1Hz of (a) T-h system and (b) T-l system.

As the volume fraction of filler increases, the loss modulus of silicone rubber correspondingly rises, aligning with the trend observed in the storage modulus of silica-enhanced silicone rubber. Nevertheless, the loss modulus remains an order of magnitude lower than the energy storage modulus. When silica volume fraction is above 12.00%, the loss modulus increases first and then decreases, showing the energy dissipation effect during the failure of the three-dimensional filler network, which also indicates that a more perfect three-dimensional filler network is formed when the volume content of silica reaches about 12%.

Reference

1. Xiong, Y.; Shen, S.; Kang, M.; Wang, Z.; Lu, A. Effect of fluorescence labeling on mechanical properties of silica filled silicone rubber. *Polymer* **2020**, *208*, 122904. https://doi.org/10.1016/j.polymer.2020.122904.
